# Early childhood caries–related complications in pediatric dental emergencies: a 7-year retrospective study from Romania

**DOI:** 10.1007/s00784-026-06968-8

**Published:** 2026-06-04

**Authors:** Rebeca Daniela Marton, Abel Emanuel Moca, Petra Opriș, Raluca Iurcov, Mihai Juncar

**Affiliations:** Department of Dental Medicine, University of Oradea, Oradea, Romania

**Keywords:** Early childhood caries, Pediatric dental emergencies, Dental abscess, Furcation periodontitis, Preventive dentistry

## Abstract

**Objectives:**

Early childhood caries (ECC) is a highly prevalent and largely preventable disease that may lead to severe complications requiring emergency dental care. This study aimed to retrospectively analyze the presentation patterns, clinical characteristics, and management of ECC-related complications among children presenting to a pediatric dental emergency service over a seven-year period.

**Materials and methods:**

A retrospective observational study was conducted using medical records from the dental emergency department of the County Clinical Emergency Hospital in Oradea, Romania, covering the period January 2019 to December 2025. Children aged 0–71 months presenting with ECC-related complications were included. Demographic variables, diagnosis, tooth location, and emergency treatment were recorded. Descriptive statistics were applied, and associations between variables were assessed using chi-square and Kruskal–Wallis tests.

**Results:**

A total of 1490 children were included (mean age: 4.81 ± 1.11 years). Furcation periodontitis (38.1%) and pulpitis (36.8%) were the most frequent diagnoses, followed by dental abscesses (22.4%). Mandibular posterior teeth were predominantly affected (59.5%). Children from rural areas presented proportionally more often with advanced complications (*p* = 0.009). Significant associations were observed between diagnosis and tooth location (*p* < 0.001), as well as between diagnosis and age (*p* < 0.001). A marked reduction in emergency presentations was observed during 2020–2021, followed by a progressive increase after 2022.

**Conclusions:**

ECC-related complications remain a major cause of pediatric dental emergency visits, with most children presenting at advanced stages of disease.

**Clinical relevance:**

These findings highlight delayed access to preventive dental care and underscore the need for early, community-based preventive strategies to reduce severe ECC complications and emergency dental service utilization.

## Introduction

Early childhood caries (ECC) is defined by the International Association of Paediatric Dentistry (IAPD) in the Bangkok Declaration as the presence of one or more decayed (non-cavitated or cavitated lesions), missing, or filled (due to caries) surfaces in any primary tooth of a child under six years of age [1]. ECC represents one of the most prevalent oral diseases globally and exerts a substantial negative impact on children’s quality of life [2]. The domains most severely affected include symptoms and psychological well-being [3], resulting in immediate consequences such as dental pain [4]. Furthermore, untreated ECC may lead to long-term psychosocial effects, including bullying associated with caries of the anterior teeth [5], which has been linked to difficulties in emotional well-being in adulthood [6].

The factors influencing the development of ECC are numerous, and its etiology is complex. On the one hand, several sociodemographic factors have been associated with a higher prevalence of ECC, such as low socioeconomic status, low parental educational level, and larger household size [7]. Additionally, dietary factors, including daily consumption of sweets and sugar-sweetened beverages, as well as oral hygiene–related factors, such as the absence of daily toothbrushing and the use of non-fluoridated toothpaste, play a significant role in the increased prevalence of this condition [7]. Considering the early age at which ECC may occur, including in children younger than three years, breastfeeding has been investigated as a potential risk factor. Evidence suggests that nocturnal breastfeeding is associated with an increased risk of dental caries in preschool children [8]. Although the IAPD acknowledges the protective role of breastfeeding up to the age of one year, it warns that continued breastfeeding beyond 12 months may be associated with an increased risk of caries development [9].

Despite its numerous negative effects and the fact that it is a largely preventable disease [10], ECC remains largely untreated worldwide [11]. Beyond its long-term consequences, untreated ECC may lead to immediate complications, such as pulpitis, furcation periodontitis, or dental abscesses, which in turn may result in severe systemic complications that can be life-threatening [12]. These acute complications are often the reason for a child’s first dental visit, with pain being one of the main drivers for seeking dental care [13].

In Romania, according to a report of the National Institute of Statistics on oral health, the prevalence of caries affecting primary teeth among children aged 1 to 9 years is 48.2%, exceeding the European average of 33.6% and ranking Romania first among European Union countries in this regard [14]. The underlying reasons are multiple and not yet fully elucidated; however, evidence suggests that although parents generally demonstrate relatively good knowledge and appropriate attitudes toward dental care, their oral health–related practices remain suboptimal [15]. In addition, regarding pediatric presentations to emergency dental services, a study conducted in Oradea, Romania, published in 2024 and analyzing emergency dental care data from 2022 to 2023, revealed that nearly 20% of all patients presenting to emergency services were children aged between 2 and 9 years [16]. Nevertheless, data describing the actual burden of pediatric cases presenting to dental emergency services remain limited. To date, no studies have been identified in the scientific literature that specifically analyze emergency dental presentations of children under six years of age for the urgent management of ECC-related complications. Moreover, no retrospective studies evaluating such data over a period longer than five years have been reported.

Therefore, the aim of this study was to retrospectively analyze the presentation of children with ECC to the dental emergency department of the County Clinical Emergency Hospital in Oradea, Romania, over the entire period for which digital records were available, from January 2019 to December 2025. The analysis focused on ECC-related complications, including pulpitis, furcation periodontitis, dental abscesses, and other complications associated with ECC.

## Materials and methods

### Study design and data collection

This study was designed as a retrospective observational study based on data collected from the dental emergency service of the County Clinical Emergency Hospital in Oradea, located in the North-West region of Romania, and was reported in accordance with the Strengthening the Reporting of Observational Studies in Epidemiology (STROBE) guidelines. The hospital is a university-affiliated institution associated with the University of Oradea. The dental emergency service provides free-of-charge emergency dental care for both pediatric and adult patients presenting with acute dental conditions.

Medical records of patients who presented to the dental emergency service between 1 January 2019 and 31 December 2025 were reviewed. For the construction of the study database, only pediatric patients aged between 0 and 71 months (corresponding to 5 years and 11 months) were included. Eligible patients were those who presented with complications affecting the primary dentition related to ECC, such as pulpitis, furcation periodontitis, or dental abscess, and who were residents of Romania.

**Fig. 1:**
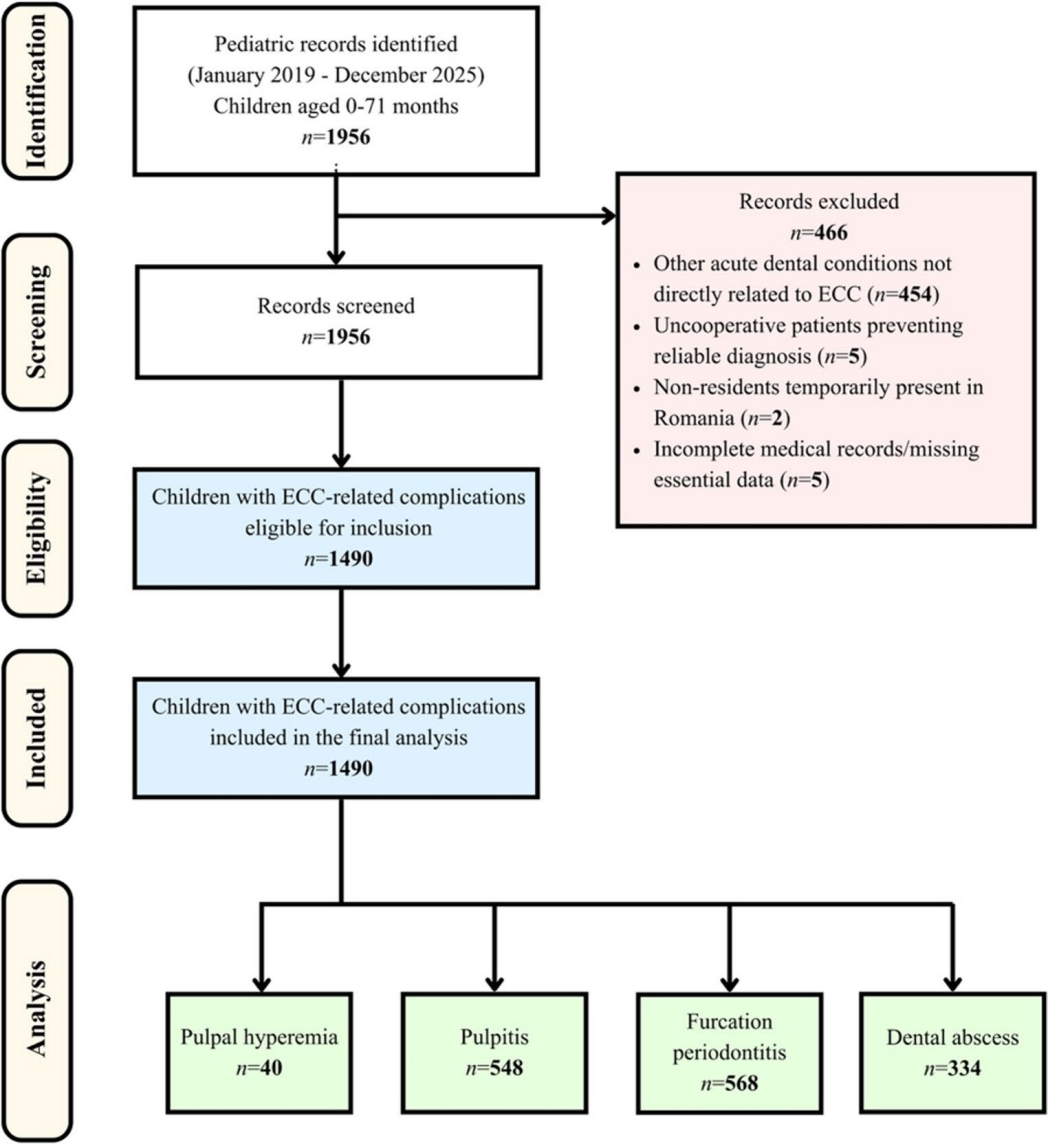
Flow diagram of the record selection process for the retrospective analysis of ECC-related complications in pediatric dental emergencies (2019–2025)

Patients were excluded if they presented with other acute dental conditions not directly related to ECC (e.g., gingival emergencies or dental trauma), if they were uncooperative to an extent that prevented the determination of a reliable diagnosis, if they were non-residents temporarily present in Romania, or if their medical records were incomplete or lacked essential data (e.g., age, living environment, or diagnosis) (Fig. 1).

Between July 2025 and January 2026, one of the study authors (M.R.D.) retrospectively extracted all available data corresponding to eligible patients, starting from the first digital registration of dental emergency cases in January 2019 until the end of December 2025. Data were collected using a standardized data extraction form and were subsequently reviewed to ensure completeness and internal consistency.

The following variables were recorded: year and month of presentation, patient age, sex (male/female), living environment (urban/rural), dental diagnosis (pulpal hyperemia, pulpitis, furcation periodontitis, dental abscess), affected primary tooth, and type of emergency treatment provided (consultation, temporary dressing, or drainage).

Emergency treatment was performed in accordance with the local clinical protocol of the dental emergency department. Drainage associated with antibiotic therapy was indicated in cases presenting with acute furcation periodontitis with swelling or dental abscess with localized or diffuse fluctuant swelling. The antibiotic of choice was amoxicillin with clavulanic acid, administered according to the patient’s weight and age. Sedative dressing with zinc oxide-eugenol (ZOE) was applied in cases of pulpitis or early furcation periodontitis without signs of acute exacerbation, as a temporary measure to relieve pain pending definitive treatment.

Diagnoses were established based on a combination of clinical and radiographic findings, in accordance with the standardized diagnostic criteria embedded in the electronic registration system used by the emergency department. The operational definitions applied for each diagnostic category are presented in Table 1 [17].

**Table 1:** Operational definitions of diagnostic categories used in the study

Diagnosis	Clinical criteria	Radiographic criteria
Pulpal hyperemia	Mild, transient pain to thermal or tactile stimuli; no spontaneous pain	No periapical changes
Pulpitis	Spontaneous pain or prolonged response to stimuli; possible mild swelling	No or minimal periapical changes
Furcation periodontitis	Tenderness to percussion; possible sinus tract or localized swelling	Periapical radiolucency
Dental abscess	Localized fluctuant or diffuse swelling; possible fever or regional lymphadenopathy	Periapical radiolucency with or without alveolar bone destruction

### Statistical analysis

Statistical analysis was performed using IBM SPSS Statistics version 25 (IBM Corp., Armonk, NY, USA) and Microsoft Excel/Word 2025 (Microsoft Corporation, Redmond, WA, USA). Quantitative variables were summarized using means and standard deviations, as well as minimum and maximum values. The distribution of age across diagnostic and tooth localization groups was assessed using the non-parametric Kruskal–Wallis H test, with epsilon squared (ε²) reported as the corresponding effect size. Categorical variables were expressed as absolute frequencies and percentages. Associations between categorical variables, including diagnosis, gender, place of residence, tooth localization, and treatment type were evaluated using the chi-square (χ²) test, with Cramér’s V reported as the effect size measure. A two-sided p-value < 0.05 was considered statistically significant. Additionally, a binary logistic regression analysis was performed to identify independent predictors of advanced ECC-related complications (furcation periodontitis and dental abscess), with living environment, sex, age, and year of presentation as covariates. Results are expressed as odds ratios (OR) with 95% confidence intervals (CI).

### Ethical considerations

Despite the retrospective nature of the study, ethical approval was obtained from the Ethics Committee of the University of Oradea (IRB No. 9970/23.06.2025). No patient names or identifiable personal data were recorded during data collection. All procedures were conducted in accordance with the principles of the Declaration of Helsinki (1964) and its subsequent amendments. As the study was conducted within a university-affiliated hospital setting, parents or legal guardians provided informed consent at the time of presentation, allowing the anonymized use of their children’s medical data for future research purposes.

## Results

The overall descriptive characteristics of the study population are summarized in Table 2. A total of 1490 children with ECC-related complications were included in the analysis. The study population showed a slight male predominance, with 798 males (53.6%) and 692 females (46.4%). Most children originated from urban environments (*n* = 911, 61.1%), while 579 (38.9%) were from rural areas. The mean age was 4.81 ± 1.11 years (range: 1–6 years). Furcation periodontitis was the most frequent diagnosis (*n* = 568, 38.1%), followed by pulpitis (*n* = 548, 36.8%) and dental abscesses (*n* = 334, 22.4%). Regarding tooth location, mandibular posterior teeth were most affected (*n* = 887, 59.5%), followed by maxillary posterior teeth (*n* = 461, 30.9%). Drainage associated with antibiotic therapy was the most frequently applied emergency treatment (*n* = 902, 60.5%).

**Table 2:** Overall descriptive characteristics of the study population (2019–2025)

Variable	*n*	%
Sex		
Male	798	53.6
Female	692	46.4
Living environment		
Urban	911	61.1
Rural	579	38.9
Age (years)		
Mean ± SD^a^	4.81 ± 1.11	
Range (min–max)	1–6	
Diagnosis		
Hyperemia	40	2.7
Pulpitis	548	36.8
Furcation periodontitis	568	38.1
Dental abscess	334	22.4
Tooth location		
FS^b^	135	9.1
FI^c^	7	0.5
LS^d^	461	30.9
LI^e^	887	59.5
Emergency treatment		
Drainage + ATB^f^	902	60.5
Sedative dressing	588	39.5
TOTAL	1490	100

^a^ SD – Standard Deviation; ^b^ FS - maxillary anterior teeth; ^c^ FI - mandibular anterior teeth; ^d^ LS - maxillary posterior teeth; ^e^ LI - mandibular posterior teeth; ^f^ ATB – antibiotic

As shown in Table 3, ECC-related complications accounted for the majority of pediatric dental emergency visits among children aged 0–71 months throughout the entire study period, representing 76.6% of all presentations overall (1490/1946). The proportion of ECC-related cases increased progressively over time, from 64.6% in 2019 to 87.9% in 2025, suggesting a growing burden of ECC-related complications relative to other causes of pediatric dental emergencies. The lowest proportions were recorded in 2019, while the highest was observed in 2025. Non-ECC cases, which included conditions such as dental trauma and gingival emergencies, accounted for 23.4% of all presentations overall, with a declining proportional trend observed in the later years of the study period.

**Table 3:** Annual distribution of total pediatric dental emergency visits by ECC-related cases and non-ECC cases (2019–2025)

Year	Total emergency visits (0–71 months) (*n*)	ECC-related cases		Non-ECC cases	
		*n*	%	*n*	%
2019	367	237	64.6	130	35.4
2020	191	146	76.4	45	23.6
2021	147	111	75.5	36	24.5
2022	295	232	78.6	63	21.4
2023	305	230	75.4	75	24.6
2024	336	266	79.2	70	20.8
2025	305	268	87.9	37	12.1
Total	1946	1490	76.6	456	23.4

The year-by-year distribution of demographic and clinical characteristics is presented in Table 4. The number of children presenting with ECC-related complications varied across the study period, with a marked decrease in 2020 and 2021, followed by a progressive increase from 2022 onward.

**Table 4:** Year-by-year distribution of demographic and clinical characteristics of children with ECC-related complications presenting to the dental emergency department

Variable	2019		2020		2021		2022		2023		2024		2025	
	*n*	%	*n*	%	*n*	%	*n*	%	*n*	%	*n*	%	*n*	%
Sex														
Male	143	60.3	77	52.7	60	54.1	114	49.1	115	50.0	148	55.6	141	52.6
Female	94	39.7	69	47.3	51	45.9	118	50.9	115	50.0	118	44.4	127	47.4
Living environment														
Urban	119	50.2	90	61.6	76	68.5	173	74.6	144	62.6	149	56.0	160	59.7
Rural	118	49.8	56	38.4	35	31.5	59	25.4	86	37.4	117	44.0	108	40.3
Age (years)														
Mean ± SD^a^	4.7 ± 1.17		4.75 ± 1.25		4.86 ± 1.33		4.82 ± 1.00		4.77 ± 1.08		4.88 ± 1.01		4.85 ± 1.08	
Range (min–max)	2–6		1–6		1–6		1–6		2–6		1–6		2–6	
Diagnosis														
Hyperemia	0	0	4	2.7	1	0.9	11	4.7	7	3.1	8	3.0	9	3.4
Pulpitis	108	45.6	56	38.4	42	37.8	97	41.8	76	33.0	87	32.7	82	30.6
Furcation periodontitis	81	34.2	58	39.7	50	45.0	86	37.1	94	40.9	104	39.1	95	35.4
Dental abscess	48	20.3	28	19.2	18	16.3	38	16.4	53	23.0	67	25.2	82	30.6
Tooth location														
FS^b^	19	8.0	21	14.4	15	13.5	18	7.8	21	9.2	20	7.5	21	7.8
FI^c^	1	0.4	1	0.7	0	0.0	0	0.0	1	0.4	2	0.8	2	0.7
LS^d^	79	33.3	52	35.6	42	37.8	71	30.6	70	30.4	73	27.4	74	27.6
LI^e^	138	58.2	72	49.3	54	48.7	143	61.6	138	60.0	171	64.3	171	63.7
Emergency treatment														
Drainage + ATB^f^	129	54.4	86	58.9	68	61.3	124	53.4	147	63.9	171	64.3	177	66.0
Sedative dressing	108	45.6	60	41.1	43	38.7	108	46.6	83	36.1	95	35.7	91	34.0
TOTAL	237	100	146	100	111	100	232	100	230	100	266	100	268	100

^a^ SD – Standard Deviation; ^b^ FS - maxillary anterior teeth; ^c^ FI - mandibular anterior teeth; ^d^ LS - maxillary posterior teeth; ^e^ LI - mandibular posterior teeth; ^f^ ATB – antibiotic

Across all years, a slight male predominance was consistently observed; however, no statistically significant association was found between year of presentation and sex (*p* = 0.355). In contrast, the proportion of children originating from urban environments changed significantly over time (*p* < 0.001), with a noticeable increase between 2021 and 2023. Mean age remained relatively stable throughout the study period, ranging from 4.7 to 4.9 years.

Regarding diagnosis, pulpitis and furcation periodontitis were the most frequent conditions each year; however, a statistically significant association was observed between year and diagnosis (*p* < 0.001), reflecting temporal changes in the distribution of ECC-related complications. Notably, a gradual increase in the proportion of dental abscesses was observed in the later years, reaching 30.6% in 2025.

Tooth involvement was predominantly lateral, especially in the mandibular posterior region, which consistently accounted for more than half of the affected teeth each year. No statistically significant association was identified between year of presentation and tooth location (*p* = 0.17), indicating a stable pattern of tooth involvement over time.

In terms of emergency management, drainage associated with antibiotic therapy represented the most frequently applied treatment across all years, with a progressive increase from 54.4% in 2019 to 66.0% in 2025. This trend was supported by a statistically significant association between year and type of emergency treatment (*p* = 0.022), suggesting an increase in the severity of presentations over time.

As shown in Table 5, the distribution of ECC-related diagnoses was similar between male and female patients. Pulpitis, furcation periodontitis, dental abscesses, and hyperemia were comparably represented in both genders, with no statistically significant differences observed (*p* = 0.355, Cramér’s V = 0.047). Overall, these findings indicate that the type of ECC-related complication leading to emergency presentation was not influenced by the child’s gender.

**Table 5:** Association between diagnosis and gender (2019–2025)

Diagnosis	Male		Female		Total		p
	*n*	%	*n*	%	*n*	%	
Hyperemia	21	52.5	19	47.5	40	100	0.355
Pulpitis	293	53.5	255	46.5	548	100	
Furcation periodontitis	304	53.5	264	46.5	568	100	
Dental abscess	178	53.3	156	46.7	334	100	
Total	798	53.5	692	46.5	1490	100	

Table 6 presents the association between diagnosis and living environment. A statistically significant difference was observed in the distribution of ECC-related diagnoses between children from urban and rural areas (*p* = 0.009, Cramér’s V = 0.088). Pulpitis was more frequently reported among children from urban settings, accounting for 65.1% (357/548) of cases, compared with 34.9% (191/548) among rural children. In contrast, more severe complications showed a higher proportional representation in rural areas, including furcation periodontitis (41.7% rural vs. 58.3% urban) and dental abscesses (39.8% rural vs. 60.2% urban). Although urban cases remained numerically predominant overall, the relative burden of advanced ECC-related complications was higher among children residing in rural areas, suggesting differences in disease presentation associated with residential background.

**Table 6:** Association between diagnosis and living environment (2019–2025)

Diagnosis	Urban		Rural		Total		p
	*n*	%	*n*	%	*n*	%	
Hyperemia	22	55	18	45	40	100	0.009
Pulpitis	357	65.1	191	34.9	548	100	
Furcation periodontitis	331	58.3	237	41.7	568	100	
Dental abscess	201	60.2	133	39.8	334	100	
Total	911	61.1	579	38.9	1490	100	

A strong and statistically significant association was observed between the type of diagnosis and tooth location (*p* < 0.001, Cramér’s V = 0.155). Across all diagnostic categories, lateral teeth were predominantly affected, particularly those located in the mandibular arch. Among children diagnosed with pulpitis, 62.4% (342/548) of cases involved mandibular lateral teeth, followed by maxillary lateral teeth in 32.8% (180/548) of cases, whereas frontal teeth accounted for less than 5% of presentations. Similarly, furcation periodontitis and dental abscesses showed a marked predominance in mandibular lateral teeth, representing 62.3% (354/568) and 51.2% (171/334) of cases, respectively. In contrast, frontal teeth were infrequently involved across all diagnoses, accounting for less than 10% of cases overall (Table 7). This distribution highlights tooth localization, particularly involvement of mandibular lateral teeth, as a key factor associated with the severity of ECC-related complications presenting as dental emergencies.

**Table 7:** Association between diagnosis and tooth location (2019–2025)

Diagnosis	FS^a^		FI^b^		LS^c^		LI^d^		Total		p
	*n*	%	*n*	%	*n*	%	*n*	%	*n*	%	
Hyperemia	8	20.0	2	0.4	12	30	20	50	40	100	< 0.001
Pulpitis	24	4.4	2	0.4	180	32.8	342	62.4	548	100	
Furcation periodontitis	55	9.7	2	0.4	157	27.6	354	62.3	568	100	
Dental abscess	48	14.4	3	0.9	112	33.5	171	51.2	334	100	
Total	135	9.1	7	0.4	461	31.0	887	59.5	1490	100	

^a^ FS - maxillary anterior teeth; ^b^ FI - mandibular anterior teeth; ^c^ LS - maxillary posterior teeth; ^d^ LI - mandibular posterior teeth

Table 8 summarizes the age distribution according to diagnosis and tooth location. A statistically significant difference in age was observed across diagnostic categories (H = 34.110, *p* < 0.001, ε²=0.021). Children presenting with hyperemia were younger (mean age: 4.10 ± 1.24 years; median: 4 years) compared with those diagnosed with pulpitis (4.84 ± 1.03 years), furcation periodontitis (4.96 ± 1.04 years), and dental abscesses (4.58 ± 1.25 years), all of which showed a median age of 5 years. Overall, more severe ECC-related complications tended to occur in slightly older children within the studied age range (1–6 years).

**Table 8:** Age distribution according to diagnosis and tooth location (2019–2025)

Variable	*n*	Age			*p*
		Mean ± SD^a^ (years)	Median (years)	Min–Max	
Diagnosis					
Hyperemia	40	4.10 ± 1.24	4	1–6	< 0.001
Pulpitis	548	4.84 ± 1.03	5	1–6	
Furcation periodontitis	568	4.96 ± 1.04	5	1–6	
Dental abscess	334	4.58 ± 1.25	5	1–6	
Tooth location					
FS^b^	135	4.32 ± 1.21	4	1–6	< 0.001
FI^c^	7	3.86 ± 1.35	4	1–6	
LS^d^	461	4.79 ± 1.05	5	1–6	
LI^e^	887	4.94 ± 1.03	5	1–6	

^a^ SD – Standard Deviation; ^b^ FS - maxillary anterior teeth; ^c^ FI - mandibular anterior teeth; ^d^ LS - maxillary posterior teeth; ^e^ LI - mandibular posterior teeth

A similarly significant age difference was observed according to tooth location (H = 116.545, *p* < 0.001, ε²=0.076). Involvement of anterior teeth was associated with younger age, particularly mandibular anterior teeth (FI), where the mean age was 3.86 ± 1.35 years. In contrast, posterior teeth, especially mandibular posterior teeth (LI), were affected in older children (mean age: 4.94 ± 1.03 years; median: 5 years), followed by maxillary posterior teeth (LS: 4.79 ± 1.05 years). These findings indicate a progressive shift toward posterior tooth involvement with increasing age.

To assess whether the observed associations between living environment, year of presentation, and diagnostic severity were independent of potential confounding factors, a binary logistic regression analysis was performed. Advanced ECC-related complications (furcation periodontitis and dental abscess) were used as the dependent variable, with living environment, sex, age, and year of presentation as independent variables. The results are presented in Table 9. After adjusting for all covariates, rural residence remained a significant independent predictor of advanced complications (OR = 1.351, 95% CI: 1.087–1.679, *p* = 0.007). Year of presentation was also a significant predictor (OR = 1.084, 95% CI: 1.031–1.141, *p* = 0.002), confirming the progressive increase in severity of presentations over time. Sex and age were not independently associated with diagnostic severity after adjustment (*p* = 0.119 and *p* = 0.530, respectively).

**Table 9:** Binary logistic regression analysis of predictors of advanced ECC-related complications (2019–2025)

Variable	OR	95% CI	*p*-value
Rural residence (vs. urban)	1.351	1.087–1.679	0.007
Male sex (vs. female)	1.182	0.958–1.460	0.119
Age (years)	1.031	0.938–1.133	0.530
Year of presentation	1.084	1.031–1.141	0.002

## Discussion

The present study provides a comprehensive seven-year analysis of pediatric presentations with complications of untreated ECC to a dental emergency service. The findings confirm that untreated ECC remains a major reason for presentation to pediatric dental emergency departments, with most children being evaluated at advanced stages of the disease. This observation is consistent with international studies reporting that pain and infection are the primary reasons prompting parents to seek emergency dental care for young children, particularly in the absence of regular preventive dental visits [18, 19].

This late presentation of children with ECC should also be interpreted in the context of the temporal framework in which access to dental emergency services occurred. The annual distribution of presentations remained relatively stable throughout most of the study period, with the exception of 2020–2021, when a marked decrease in the number of emergency visits was observed. The years 2020 and 2021 correspond to the period during which restrictive measures associated with the COVID-19 pandemic were still in effect in Romania [20], even though the dental emergency service of the County Clinical Emergency Hospital in Oradea remained operational throughout the lockdown. In this context, it is likely that these restrictions contributed to reduced attendance at dental emergency services. Fear of infection, legislative restrictions, and the postponement of dental treatments have been identified as key factors underlying this decrease in other studies as well [21]. A similar trend has been reported in the international literature. Data from various healthcare systems have documented a significant reduction in access to pediatric dental services during pandemic-related restrictions compared with pre- and post-pandemic periods. Accordingly, Yang et al. reported that the resumption of pediatric dental activity following the COVID-19 lockdown, under continued restrictions in 2020, was associated with a significantly lower number of patient presentations and with changes in both diagnostic and therapeutic profiles compared with the pre-pandemic year of 2019 [22]. Similarly, studies conducted in pediatric populations from Serbia [23], Cyprus [24], and Austria [25] reported significantly fewer emergency dental presentations during lockdown periods compared with pre- or post-lockdown intervals. However, current literature provides limited comparative data including the more recent post-pandemic years. In this regard, our observations of increased emergency presentations after 2022 add relevant evidence on the medium-term impact of the COVID-19 pandemic on the presentation of children with ECC to dental emergency services.

Beyond temporal variations in service utilization, the analysis of the types of pathology encountered at presentation provides essential insights into the clinical severity of cases evaluated in the pediatric dental emergency setting. In our study, furcation periodontitis and dental abscesses accounted for a substantial proportion of all cases, and the progressive increase in the frequency of dental abscesses in recent years indicates a growing severity of presentations. These findings are consistent with data reported in the literature, which demonstrate that untreated ECC frequently progresses to pulpal and periapical infections [26]. Similar to the results of the present study, other authors have reported that odontogenic infectious conditions, such as pulpitis, furcation periodontitis, and dental abscesses, represent some of the most common reasons for presentation to pediatric dental emergency services. Accordingly, Shqair et al. and Elalouf et al. highlighted a high prevalence of pulpal and periapical infections among pediatric dental emergencies, particularly in the context of untreated caries and delayed presentation to dental care [27, 28]. Nevertheless, some studies have identified dental trauma as the leading cause of presentation to pediatric dental emergency services, especially within specific age groups or differing organizational contexts. For example, Jung et al. reported that traumatic dental injuries constituted the most frequent reason for emergency visits in the populations analyzed, exceeding the frequency of odontogenic infections [29]. Taken together, these observations suggest that delayed presentation of children with ECC is closely associated with increased clinical severity of cases encountered in pediatric dental emergency services, underscoring the importance of early access to preventive care and regular monitoring in pediatric populations.

In addition to the clinical characteristics of presentations, the analysis of patients’ demographic features provides further insight into factors influencing the severity of ECC. The significant differences observed in our study according to living environment and age are consistent with patterns described in the literature regarding access to dental services and ECC progression. Our analysis showed that, although children from urban areas were more frequently represented among dental emergency presentations, those from rural environments presented proportionally more often with advanced stages of the disease, requiring more invasive emergency interventions. These findings are similar to those reported in previous studies, which attribute this disparity to limited access to dental care, lower socioeconomic status, and inadequate oral health education in rural populations [30, 31].

In parallel, the age-related differences identified in our study across diagnostic categories and affected tooth locations reflect the progression patterns of ECC described in the literature, according to which carious lesions appear early in anterior teeth and subsequently extend to posterior teeth as age increases [10, 26]. Our results confirm these observations and highlight both the need for community-based approaches tailored to the place of residence and the existence of a critical window for preventive intervention during the early years of life. Early ECC prevention, initiated in the first years of life through early caries risk assessment, parental education, fluoride use, and regular dental check-ups, represents an essential strategy for reducing disease progression and associated complications, as supported by international guidelines and studies [1, 32, 33].

The emergency management applied in this study reflects a common approach in resource-limited or high-demand settings, where definitive pulp treatment or extraction cannot always be performed at the time of first presentation. While drainage and antibiotic therapy are appropriate for acute odontogenic infections with signs of systemic involvement or spreading infection, the exclusive reliance on emergency management raises important concerns regarding antibiotic stewardship. Systemic antibiotics are not a substitute for definitive dental treatment, and their use without subsequent follow-up may contribute to incomplete resolution of infection and promote antimicrobial resistance. Current guidelines recommend that antibiotics should be reserved for cases with systemic signs or spreading infection, and that definitive treatment should be completed as soon as possible [34, 35].

The limitations of this study should be considered when interpreting the results. The absence of data on follow-up and definitive treatment in the present study represents a limitation, as it is unknown what proportion of children received subsequent care after the emergency visit. Then, the retrospective design entails reliance on the accuracy and completeness of existing medical records, which may introduce reporting or classification biases.The single-center nature of the study may limit the extent to which these findings can be applied to other regions or healthcare systems, particularly those with different structures of access to dental services. In addition, the lack of detailed information regarding families’ socioeconomic status, oral hygiene behaviors, history of preventive dental visits, and subsequent definitive treatments precluded a more in-depth analysis of factors influencing ECC progression. Furthermore, data extraction was performed by a single author (M.R.D.) without a secondary reviewer, which may introduce the possibility of data extraction errors, despite the use of a standardized electronic registration system with predefined diagnostic categories.

Despite these limitations, the findings provide a solid basis for future research directions. Prospective, multicenter studies incorporating detailed socioeconomic and behavioral variables could contribute to a more comprehensive understanding of the mechanisms underlying delayed presentation of children with ECC. Furthermore, evaluating the impact of early prevention programs and parent-focused educational interventions on reducing emergency dental visits represents an important avenue for future investigation. Integrating pediatric dentistry into public health strategies and developing community-based, prevention-oriented models of care may substantially reduce the burden of ECC-related complications on dental emergency services.

## Conclusions

This seven-year retrospective study shows that untreated ECC is associated with severe complications requiring pediatric dental emergency care, with most children presenting at advanced stages of disease. The findings highlight delayed access to preventive services and persistent inequalities related to age and place of residence. Strengthening early, community-based preventive strategies and parental education is essential to reduce ECC-related complications and emergency dental visits.

## Data Availability

The datasets generated and analyzed during the current study are available from the corresponding author on reasonable request.
